# Non communicable disease multimorbidity and associated health care utilization and expenditures in India: cross-sectional study

**DOI:** 10.1186/1472-6963-14-451

**Published:** 2014-10-02

**Authors:** Sanghamitra Pati, Sutapa Agrawal, Subhashisa Swain, John Tayu Lee, Sukumar Vellakkal, Mohammad Akhtar Hussain, Christopher Millett

**Affiliations:** Indian Institute of Public Health-Bhubaneswar, Public Health Foundation of India, 2nd and 3rd Floor, JSS Software Technology Park, E1/1, Infocity Road, Patia, Bhubaneswar, Odisha India; South Asian Network for Chronic Disease, Public Health Foundation of India, New Delhi, India; School of Public Health, Imperial College London, London, UK; School of Population Health, The University of Queensland, Brisbane, Australia

**Keywords:** Non-communicable disease (NCD), Multimorbidity, Health care utilization, Out-of-pocket expenditure (OOPE), WHO-SAGE, India

## Abstract

**Background:**

Non communicable disease (NCD) multimorbidity is increasingly becoming common in high income settings but little is known about its epidemiology and associated impacts on citizens and health systems in low and middle-income countries (LMICs). We aim to examine the socio-demographic distribution of NCD multimorbidity (≥2 diseases) and its implications for health care utilization and out-of-pocket expenditure (OOPE) in India.

**Methods:**

We analyzed cross-sectional nationally representative data from the World Health Organisaion Study on Global Ageing and Adult Health (WHO-SAGE), conducted in India during 2007. Multiple logistic regression was used to determine socio-demographic predictors of self-reported multimorbidity. A two part model was used to assess the relationship between number of NCDs and health care utilization including OOPE.

**Results:**

28.5% of the sample population had at least one NCD and 8.9% had NCD multimorbidity. The prevalence of multimorbidity increased from 1.3% in 18–29 year olds to 30.6% in those aged 70 years and above. Mean outpatient visits in the preceding 12 months increased from 2.2 to 6.2 and the percentage reporting an overnight hospital stay in the past 3 years increased from 9% to 29% in those with no NCD and ≥2 NCDs respectively (p <0.001).

OOPE incurred during the last outpatient visit increased from INR 272.1 (95% CI = 249.0-295.2) in respondents with no NCDs to INR 454.1 (95% CI = 407.8-500.4) in respondents with ≥2 NCDs. However, we did not find an increase in OOPE during the last inpatient visit with number of NCDs (7865.9 INR for those with zero NCDs compared with 7301.3 for those with ≥2 NCDs). For both outpatient and inpatient OOPE, medicine constitutes the largest proportion of spending (70.7% for outpatient, 53.6% for inpatient visit), followed by spending for health care provider (14.0% for outpatient, 12.2% for inpatient visit).

**Conclusion:**

NCD multimorbidity is common in the Indian adult population and is associated with substantially higher healthcare utilization and OOPE. Strategies to address the growing burden of NCDs in LMICs should include efforts to improve the management of patients with multimorbidity and reduce associated financial burden to individuals and households.

## Background

Improvements in living conditions, changing lifestyles and progress in healthcare effectiveness have led to an increase in the prevalence of non-communicable diseases (NCDs) globally [1, 2]. Much attention has been focused on the increasing burden of single or selected NCD groupings, especially diabetes and cardiovascular diseases [3]. Yet recent epidemiological studies suggest that the prevalence of NCD multimorbidity is high and increasingly the norm for patients in high income settings [4]. For example, a general practice database study conducted in Scotland found that over half of the patients (54.9%) with at least one NCD had multimorbidity [5].

International bodies and individual health systems are increasingly recognizing the importance of NCDs [6, 7]. However, most policy responses to date emphasize improving the identification and management of individual diseases [8, 9]. For example, a series of national service frameworks introduced in the UK’s National Health Service since 2000 are focused on single conditions, such as coronary heart disease. Other quality improvement strategies, including clinical guidelines and pay for performance programs, are similarly focused in single disease areas [10]. These approaches are increasingly at odds with growing information that patients with multimorbidity have higher health service utilization, health care expenditure and poorer health outcomes [11]. For example, a study of US Medicare population found that the risk of an avoidable inpatient admission and per capita medical expenditures increase dramatically with the number of chronic conditions [12–14]. The situation is not different in low and middle income countries (LMICs). In India, most of the current health programs have singular disease specific vertical approach [15, 16]. There is little information about the epidemiology of NCD multimorbidity and associated impacts on citizens and health systems in LMICs. This is an important knowledge gap given the growing burden of NCDs, the limited capacity of health systems to manage this burden and the low levels of financial protection in LMICs, resulting in a high level of out-of-pocket expenditures. Our study assessed the prevalence and predictors of NCD multimorbidity and associated impacts on health care utilization and out-of-pocket expenditure (OOPE) in India.

## Methods

### Data and sample

We used cross-sectional data from the WHO Study on Global Ageing and Adult Health (SAGE) wave 1 survey of India, 2007, carried out by the International Institute for Population Sciences, Mumbai with the technical assistance from World Health Organization. The WHO SAGE survey took representative samples of six states in India (Assam, Karnataka, Maharashtra, Rajasthan, Uttar Pradesh and West Bengal) which can be modelled to a nationally representative sample. The survey consisted of a large sample of people aged 50 years and older and a smaller comparative sample aged 18–49 years, with 12,198 respondents (4,717 men, 7,481 women) in total. The SAGE dataset is described in full elsewhere [17] and the questionnaires can be found at http://www.who.int/healthinfo/sage/cohorts/en/index2.html (accessed 18 August 2013).

The SAGE survey covers a broad range of topics, including health and its determinants, disability, subjective well-being, emotional and financial well-being, health care utilization, and health systems responsiveness. SAGE has included methodologies to improve cross-population comparability of self-reported health and well-being data through the inclusion of biomarkers, performance tests, anchoring vignettes, and additional validation studies [17]. The survey was conducted using an interviewer-administered questionnaire in the native language of the respondent using local, commonly understood terms. A total of five languages with back translation to English were used in the Indian survey to ensure accuracy and comparability.

SAGE has also worked to harmonize methods and results with a number of studies, including the U.S. Health and Retirement Study, Chinese Health and Retirement Longitudinal Study, and Longitudinal Aging Study in India [17]. For further details refer to the SAGE Wave 1 survey manual and questionnaires (available at http://www.who.int/healthinfo/sage/cohorts/en/index2.html).

For the purpose of the study, we included respondents aged ≥18 years, and excluded those with missing values for outcome and independent variables (9.2% of the sample). As some respondents did not provide a response for out-of-pocket expenditure for their last visit, our estimations were based on samples, who answered positively on this question, but included those who replied that their treatment cost was free. In addition, to lessen the influence of outliers, we remove observations with the highest 0.5% of outpatient/inpatient out-of-pocket spending.

### Variables

#### Outcome measures

Our main outcome measure was the number of NCDs reported by the respondents. Respondents were asked if they had been diagnosed with any of the following NCDs: angina, arthritis, asthma, cataract, diabetes (excluding diabetes associated with a pregnancy), stroke, chronic lung disease, hypertension and depression. The question asked was, “Have you ever been told by a health professional that you have . . . ?”, or “Have you ever been diagnosed with . . . ?” for each health condition. We defined multimorbidity as the presence of two or more above listed conditions. No standard approach for the measurement of multimorbidity exists, and selection and definition of morbidities to include in a study is inevitably partly subjective and dependent on the data available [5].

Respondents were asked about their utilization of outpatient (number of visits in the past 12 months) and hospital care (whether or not overnight stay in the past 3 years, and number of stays in past 12 months). Details about OOPE for health care provider fees, medicines, diagnostic tests and transport costs during their last outpatient visit and last hospital stay were elicited from respondents, in Indian Rupees (INR).

#### Predictor variables/covariates

Socio-economic and demographic factors included in our analysis were age groups (18–29, 30–39, 40–49, 50–59, 60–69, 70+ years); gender; caste/tribe status (general, scheduled caste and tribe, other backward classes); marital status (married, not married), education (primary school or less, secondary school completed, tertiary or higher education); location (rural, urban); state (Assam, Karnataka, Maharashtra, Rajasthan, Uttar Pradesh, West Bengal), quintiles of household wealth/assets, (Q1 lowest to Q5 highest) and health insurance status (with/without health insurance). The household wealth variable provided in the dataset was derived using WHO standard approach to estimating permanent income from survey data on household ownership of durable goods, neighborhood and dwelling characteristics, and access to water, sanitation, electricity etc. [18].

### Statistical analysis

We used multiple logistic regression to determine socioeconomic and demographic predictors of having any chronic condition or multimorbidity. We used a two-part model to assess the association between NCDs (coded as a continuous variable begins from zero) and health care utilization. In this model, we estimated whether the respondent had any outpatient or inpatient visit (binary response) using a logistic model, and estimated the number of outpatient visits or hospitalization days (count response) using a negative binomial model. We presented adjusted odds ratio (AOR) from logistic model, and coefficient from negative binomial model.

We estimated the average amount of OOPE by number of NCDs. We also presented the proportion of OOPE by categories of spending.

We tested for multicollinearity for covariates adjusted for in our analysis. The multicollinearity diagnostics (VIF) were all less than 5, indicating that the assumption of reasonable independence among predictor variables was met. Sampling weights were used to account for the complex, multi-stage design of the SAGE survey. We performed the statistical analyses using Stata 13.1 (StataCorp, College Station, Texas).

### Ethics approval

The WHO SAGE study received human subjects testing and ethics council approval from research review boards local to each participating site, and from the WHO Ethical Review Committee. The study was approved by Institutional Ethical Committee, Indian Institute of Public Health-Bhubaneswar. Informed consent was obtained from each respondent prior to interview and examination.

## Results

We analyzed data from 10,973 respondents. Table 1 presents respondents’ socioeconomic and demographic characteristics of the respondents. The median age of the respondents was 40 years (IQR = 30-49). There were about equivalent proportion of female and male respondent, 82.1% were married, 61.5% of the respondents have educational attainment of primary school completed or less, and 25.7% were residing in urban area.

**Table 1:** **Distribution of NCDs across socio demographic characterisitcs**

Characteristics	N (weighted %)	Zero NCD (%, 95% CI)	One NCD (%, 95% CI)	More than 2 NCDs (%, 95% CI)	AOR for having any NCD	AOR for having multimorbidity
**Age**						
18-29	1565 (24.0)	89.8 (87.6, 92.0)	9.0 (6.8, 11.1)	1.3 (0.6, 1.9)	1.00 (Ref)	1.00 (Ref)
30-39	1619 (25.2)	78.3 (75.4, 81.2)	16.9 (14.2, 19.6)	4.8 (3.4, 6.2)	2.67 (1.97, 3.62)	4.11 (2.18, 7.74)
40-49	1378 (26.0)	68.1 (64.3, 71.8)	23.2 (20.1, 26.4)	8.7 (6.5, 10.9)	4.53 (3.40, 6.03)	7.87 (4.25, 14.59)
50-59	2868 (12.1)	57.2 (54.4, 60.0)	26.3 (23.7, 28.9)	16.5 (14.0, 18.9)	7.05 (5.42, 9.16)	16.15 (8.83, 29.54)
60-69	2185 (7.7)	47.1 (43.8, 50.5)	31.6 (28.2, 35.1)	21.3 (18.4, 24.1)	10.91 (8.24, 14.45)	23.56 (13.08, 42.44)
70+	1358 (5.1)	38.6 (33.9, 43.3)	30.8 (27.0, 34.5)	30.6 (25.7, 35.5)	15.27 (11.06, 21.09)	39.15 (20.72, 73.98)
**Gender**						
Male	4242 (50.9)	71.7 (68.9, 74.5)	19.5 (17.2, 21.7)	8.8 (7.4, 10.2)	1.00 (Ref)	1.00 (Ref)
Female	6731 (49.1)	71.2 (69.4, 73.0)	19.7 (18.0, 21.5)	9.1 (8.1, 10.1)	1.15 (0.96, 1.39)	1.19 (0.97, 1.47)
**Location**						
Rural	8180 (74.3)	72.3 (70.5, 74.2)	19.3 (17.8, 20.8)	8.3 (7.3, 9.3)	1.00 (Ref)	1.00 (Ref)
Urban	2793 (25.7)	68.9 (65.0, 72.8)	20.4 (17.5, 23.3)	10.7 (8.7, 12.7)	0.97 (0.78, 1.19)	1.03 (0.79, 1.34)
**State**						
Assam	1106 (5.6)	73.7 (69.2, 78.1)	18.5 (14.8, 22.3)	7.8 (5.2, 10.3)	1.00 (Ref)	1.00 (Ref)
Karnataka	1538 (12.0)	57.8 (52.2, 63.4)	24.8 (20.8, 28.7)	17.4 (13.8, 21.1)	1.70 (1.18, 2.45)	2.38 (1.36, 4.14)
Maharashtra	1958 (20.5)	71.3 (67.2, 75.3)	19.5 (16.5, 22.5)	9.2 (7.5, 10.9)	1.01 (0.73, 1.39)	0.99 (0.63, 1.56)
Rajasthan	2191 (12.2)	81.7 (78.2, 75.2)	13.6 (11.3, 15.9)	4.7 (3.1, 6.3)	0.57 (0.41, 0.81)	0.52 (0.31, 0.87)
Uttar Pradesh	2167 (32.9)	72.5 (69.3, 75.7)	20.1 (17.2, 23.0)	7.4 (5.6, 9.1)	1.03 (0.74, 1.44)	0.84 (0.52, 1.35)
West Bengal	2013 (16.8)	71.2 (66.3, 76.2)	19.8 (16.5, 23.1)	9.0 (6.2, 11.8)	1.09 (0.77, 1.55)	1.06 (0.61, 1.84)
**Caste/tribe status**						
General	6401 (60.9)	75.1 (69.7, 80.6)	16.4 (13.8, 19.0)	8.7 (5.5, 11.9)	1.00 (Ref)	1.00 (Ref)
Scheduled tribes	752 (6.4)	77.5 (74.3, 80.7)	16.4 (13.8, 19.0)	6.1 (4.6, 7.7)	0.88 (0.63, 1.23)	1.02 (0.67, 1.56)
Scheduled castes	1946 (19.2)	59.8 (54.7, 64.9)	24.9 (21.1, 28.7)	15.2 (12.3, 18.2)	0.76 (0.60, 0.98)	0.75 (0.52, 1.06)
Other backward class	1874 (13.5)	71.7 (69.6, 73.9)	19.8 (18.0, 21.6)	8.5 (7.3, 9.6)	1.16 (0.80, 1.68)	0.94 (0.54, 1.63)
**Marital status**						
Married	8522 (82.1)	71.5 (69.6, 73.4)	19.7 (18.2, 21.2)	8.8 (7.8, 9.9)	1.00 (Ref)	1.00 (Ref)
Not married	2451 (17.9)	71.3 (67.8, 74.8)	19.2 (16.4, 22.1)	9.5 (7.9, 11.1)	1.08 (0.87, 1.34)	0.95 (0.75, 1.21)
**Education**						
Primary school or less	7797 (61.5)	69.9 (68.1, 71.8)	20.4 (18.9, 22.0)	9.6 (8.6, 10.6)	1.00 (Ref)	1.00 (Ref)
Secondary School	1369 (15.7)	74.5 (70.7, 78.2)	17.2 (14.1, 20.3)	8.4 (6.0, 10.7)	1.12 (0.89, 1.41)	1.43 (1.03, 1.98)
Tertiary or higher	1807 (22.8)	73.5 (69.7, 77.2)	19.0 (15.7, 22.4)	7.5 (5.6, 9.4)	1.12 (0.87, 1.43)	1.15 (0.86, 1.55)
**Household wealth**						
Q1 (low)	1959 (20.6)	74.8 (71.6, 78.0)	18.4 (15.7, 21.2)	6.8 (5.0, 8.5)	1.00 (Ref)	1.00 (Ref)
Q2	2115 (21.3)	75.1 (72.2, 77.9)	17.2 (14.4, 19.9)	7.8 (5.7, 9.8)	0.94 (0.74, 1.19)	1.09 (0.72, 1.67)
Q3	2095 (19.8)	70.4 (66.5, 74.3)	20.5 (17.3, 23.8)	9.1 (7.1, 11.0)	1.22 (0.95, 1.56)	1.24 (0.88, 1.74)
Q4	2315 (18.0)	68.3 (64.9, 71.7)	21.0 (17.7, 24.3)	10.7 (8.6, 12.7)	1.20 (0.93, 1.54)	1.29 (0.90, 1.84)
Q5 (high)	2489 (20.2)	68.1 (64.9, 71.3)	21.2 (18.4, 24.1)	10.7 (9.0, 12.4)	1.29 (0.99, 1.68)	1.35 (0.94, 1.95)
**Insurance status**						
With insurance	443 (3.3)	61.9 (53.8, 70.0)	25.6 (19.0, 32.1)	12.5 (6.7, 18.3)	1.00 (Ref)	1.00 (Ref)
Without insurance	10530 (96.7)	71.8 (70.0, 73.5)	19.4 (18.0, 20.8)	8.8 (7.9, 9.7)	0.96 (0.65, 1.42)	1.24 (0.68, 2.25)

### Socio-demographic predictors of NCD multimorbidity

The socio-economic and demographic characteristics of the study population by the number of NCDs are also presented in Table 1. The mean number of NCDs in the sample was 0.42 with 28.5% (95% CI = 26.8%-30.3%) hadany NCD, and 8.9% (95% CI = 8.0% -9.8%) had multimorbidity. The mean number of NCDs increased with age, in urban people and with increasing household income but did not significantly differ by gender or education. The prevalence of NCD multimorbidity increased substantially with age, from 1.3% (95% CI = 0.7-1.9%) in 18–29 year olds to 30.6% (95% CI = 25.7-35.5%) in those aged 70 years and above, AOR = 39.2 (95% CI = 20.7-74.0, for those aged 70 years and above compared to those aged 18–29 years). The prevalence of NCD multimorbidity increased substantially with increasing household wealth, from 6.8% (95% CI = 5.0%-8.5%) in the lowest wealth quintile to 10.7% (95% CI = 9.0%-12.4%) in the highest wealth quintile.

### Health care utilization by number of NCDs

**Figure 1:**
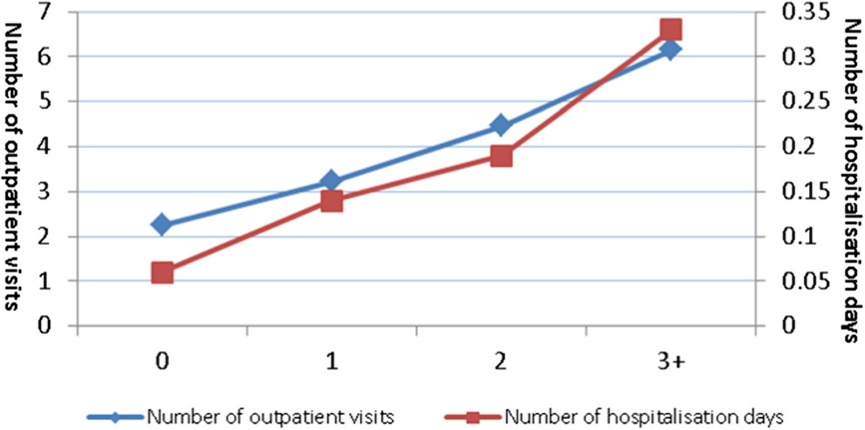
**Association between number of NCDs and health care utilisation.**

NCD multimorbidity is associated with greater healthcare utilization in primary and secondary care (Figure 1, Table 2). The percentage of participants reporting having any outpatient visits in the last year increased from 71% in those with no NCDs to 83% in those with 3+ NCDs (AOR = 1.55, p-value <0.0001). The mean number of visits to an outpatient department in the preceding 12 months increased from 2.24 in respondents with no NCDs to 6.16 in those with 3+ NCDs (regression coefficient = 0.28, p-value <0.0001). The percentage of participants reporting an overnight hospital stay in the past 3 years increased from 9% in those with no NCDs to 29% in those with 3+ NCDs (AOR = 1.59, p-value <0.0001). The mean number of stays in hospital in the past year increased from 0.06 in those with no NCDs to 0.33 in those with 3+ NCDs (regression coefficient = 0.49, p-value < 0.0001). Overall, our results suggested a positive association between number of NCDs and health care utilization for both outpatient and inpatient services.

**Table 2:** **Association between NCDs and health care utilization**

	0	1	2	3+	Coefficient for number of NCDs	P-value
**Outpatient visits**						
Any outpatient visit	0.71	0.84	0.88	0.83	1.55	<0.0001
Number of outpatient visit	2.24	3.22	4.46	6.16	0.28	<0.0001
**Inpatient visits**						
Any inpatient visit	0.09	0.15	0.23	0.29	1.59	<0.0001
Number of hospitalisation days	0.06	0.14	0.19	0.33	0.49	<0.0001

### Out-of-pocket expenditure (OOPE) by number of NCDs

The average out-of-pocket spending for outpatient and inpatient service by NCDs are presented in Table 3. We also presented the proportion of out-of-pocket spending by type of service. The OOPE incurred during the last outpatient visit increased from INR 272.1 (95% CI = 249.0-295.2) in respondents with no NCDs to INR 454.1 (95% CI = 407.81-500.35) in respondents with 2+ NCDs. However, we did not find inpatient OOPE during the last visits increased with number of NCDs (7865.9 INR for those with zero NCDs compared with 7301.3 for those with 2+ NCDs). For both outpatient and inpatient OOPE, medicine constitutes the largest proportion of spending (70.7% for outpatient, 53.6% for inpatient visit), followed by spending for health care provider (14.0% for outpatient, 12.2% for inpatient visit). There is also a non-trivial proportion of OOPE on transport (8.3% for outpatient and 11.8% for inpatient visit), and medical test (4.4% for outpatient and 10.5% for inpatient service). This pattern of spending did not alter significantly for patients with different number of NCDs.

**Table 3:** **NCDs and out-of-pocket expenditure by categories**

Number of NCDs	Outpatient					
	Total OOPE (95% CI)	Health care provider (%)	Medicines (%)	Medical test (%)	Transport (%)	Others (%)
*All*	308.9 (259.59, 328.27)	14.03%	70.71%	4.42%	8.30%	2.54%
*0*	272.07 (248.95, 295.18)	13.19%	73.55%	3.22%	7.57%	2.47%
*1*	356.79 (318.41, 395.17)	15.76%	66.23%	5.83%	9.38%	2.81%
*2+*	454.08 (407.81, 500.35)	16.00%	61.05%	9.55%	10.90%	2.50%
**Number of NCDs**	**Inpatient**					
	**Total OOPE (95% CI)**	**Health care provider (%)**	**Medicines (%)**	**Medical test (%)**	**Transport (%)**	**Others (%)**
*All*	7483.6 (6486.1, 8481.1)	12.16%	53.62%	10.49%	11.82%	11.92%
*0*	7864.9 (6215.5, 9514.4)	12.06%	55.92%	9.24%	11.68%	11.10%
*1*	6847.68 (5121.7, 8573.7)	10.56%	52.36%	12.01%	12.77%	12.30%
*2+*	7301.33 (5234.6, 9368.1)	14.47%	49.21%	11.80%	10.97%	13.56%

## Discussion

NCD multimorbidity is increasingly common globally with growing implications for the management of individual patients, assessment of disease burden in populations and health system efficiency and effectiveness [5, 19]. However, very little research has been undertaken in LMICs, where 80% of the burden of NCDs falls [20]. Our study reveals that more than one in four adults in India has at least one NCD, with 8.9% having two or more NCDs. We found no significant difference in the prevalence of multimorbidity across gender or educational attainment. Multimorbidity appears to be more common in affluent households and there is a dramatic rise in prevalence with age, increasing from 1.3% in the youngest group (18–29 years) to 30.6% in the oldest (≥70 years).

The prevalence of multimorbidity in our study appears to be lower than that reported in high income countries. A study conducted in Spain in 2007 has shown that 42% of the registered population aged 14 years and above had at least one chronic condition, with almost one quarter having multimorbidity [21]. Another study undertaken in a family practice settings in Canada has reported nine out of ten patients to have more than one chronic condition [22]. It should be noted that most of these studies have been conducted in primary care practice populations, using clinical and administrative databases [22–25]. The prevalence of multimorbidity when estimated in general population surveys has been found to be lower than when examined using clinical databases [26], partly due to the more limited numbers of conditions (only eight diseases in our study) included in these analyses. Thus, estimation studies preferably in primary care settings using broad nosological spectrum of chronic conditions would be insightful.

Our study confirms previous findings from high income countries that multimorbidity increases with age [21–23]. Available data has not demonstrated consistent associations between gender and multimorbidity, with some studies indicating a higher prevalence in women while others found similar results in men [24, 27, 28]. Recent studies emphasize the importance of examining multimorbidity across life course and not just the elderly population [29]. However NCD prevalence estimates in women should be interpreted cautiously in South Asian context, where they may have lower health care seeking behavior [30]. The possible interplay of multimorbidity with social and economic deprivation has already been identified. Studies conducted in high income countries have generally found that persons with low socio-economic status (SES) are more likely to have multimorbidity when compared to their affluent counterparts [31]. However, we did not find similar evidence in our study. A study from Bangladesh has also reported the prevalence to be more in high income group [32]. This might be due to higher health literacy levels and more frequent utilization of health care facilities in higher income groups [33].

The presence of multimorbidity was associated with substantially higher levels of health care utilization, in both outpatient and hospital settings, and markedly with higher levels of OOPE in our study. These findings are consistent with those from previous studies conducted in high income settings which have identified a positive correlation between multimorbidity with health care utilization and cost [14, 34, 35]. For example, a recent Scottish study found that persons with multimorbidity were six times more likely to have an unplanned hospital admission [11]. Our study builds on previous research in India which has documented the impoverishing impact of health care costs among persons with NCDs, wherein nearly half of OOPE was incurred in the purchase of medicine, diagnostic investigations and medical appliances [36]. We too observed that more than 50% of total expenditure is spent towards getting medicines. However, out of pocket expenditure was not specific for NCDs, and might have included treatment for conditions not related to NCDs The impact of multimorbidity alone on catastrophic and impoverishing household health care spending in LMICs merits further investigation [37]. It would be interesting first to investigate, what proportion of the identified demand of chronic healthcare is being currently delivered in primary care practice and the consequential impact on OOPE if there is transfer of healthcare services from specialist setting to primary care settings.

The key strength of this study is the use of a nationally representative sample; which permits robust cross-sectional level estimates of key variables. We identify certain limitations in our study. SAGE is a cross sectional survey which limit the causal inference between multimorbidity and health care utilization and expenditure. Identification of NCDs was based on self report of a doctor diagnosis which may be biased due to a potential under or over reporting and/or poor quality diagnosis. This may result in greater under-reporting of NCDs in persons from lower socio-economic status in particular [38, 39]. However, the amount of error may not have been substantial since studies have documented that self reported prevalence produce estimates near to true prevalence and simple disease counts may have equal predictive value of morbidity burden when compared with other, more complex measurement approaches to multimorbidity [40, 41]. With the available data, it was not possible to measure the severity of the disease or its health impacts on survey participants. Utilization and OOPE data were based on self-report, and therefore subject to recall bias, and only relate to the most recent episode of care. SAGE did not include detailed questions about the presence of communicable disease and although, reasons for health care utilization were solicited from respondents, responses were generally non-specific and incomplete. Given this, we chose to include all outpatient visits or hospital admissions in these outcome measures which could have included some health care utilization that is unrelated to NCDs.

The high prevalence of NCD multimorbidity identified here underlines the importance of current efforts to strengthen health systems in many LMICs, including India. It is essential that these efforts focus on strengthening primary care, given its key role in providing continuous, well coordinated and comprehensive care to patients with complex health needs including those with multiple NCDs. This requires a shift away from current approaches, which frequently emphasize vertical, disease specific programs with primary care having a more limited role around the management of acute illness. A collaborative, patient-centerd approach accommodating multiple care processes in primary care is required. Developing clinical practice guidelines on managing multimorbidity for primary care practitioners is an important component of this new approach. Current proposals to achieve universal health coverage (UHC) in India highlight the importance of primary care strengthening but need to be cognizant of the rising burden of multimorbidity and work to strengthen health system and individual practitioner responsiveness to this challenge. The financial burden of having multiple NCDs highlights the importance of strengthening financial protection as part of universal coverage proposals. Efforts to reduce the cost of medications, which are the major source of OOPE, are already underway in India [42].

Further research is required to better understand the epidemiology of multimorbidity and associated impacts on health care utilization and costs in India and other LMIC settings. Primary care records-based prevalence studies may provide more definite estimates of the true extent of the problem. It has been hypothesized that clustering of diseases in multimorbidity could be underpinned with common etiology and thus looking at commonly occurring patterns through cluster analysis could further elucidate the dynamics of co-morbidities [43]. As cross sectional studies only reflect the diseases present at the time of data collection, and their impact on other domains is specific to this time, it would be more useful to undertake longitudinal studies to understand the progression and impact of multimorbidity over time. Furthermore, the consequences of multimorbidity on health related quality of life, poly-pharmacy, therapeutic decision making and care preferences of patients need to be investigated. The knowledge gained from such research could help in aligning current strategies along with prioritization of health services to prepare for the challenge of multimorbidity. A stronger primary care to deal with multimorbidity in a cost effective way poses a challenging task ahead for the health systems in India.

## Conclusion

Our study provides evidence on the emerging burden of NCD multimorbidity in the Indian context, highlighting the need for better recognition by physicians, health planners and policy makers. Specifically our findings indicate a need for the growing burden of multimorbidity to be considered within the context of health system planning, encompassing workforce training and quality improvement strategies, including the development of clinical guidelines and quality indicators. Our findings reinforce the importance of strengthening primary care systems in LMICs, which is the most appropriate setting for these patients to be managed, and emphasize the need to improve financial protection in these settings. Further research is required to better understand the epidemiology of multimorbidity and associated impacts on health care utilization and costs in India and other LMIC settings.
